# A conceituação dos cuidados paliativos e a importância da integridade científica no debate acadêmico

**DOI:** 10.1590/0102-311XPT078925

**Published:** 2025-07-04

**Authors:** Claudia Brito, Vanessa dos Santos Beserra

**Affiliations:** 1 Escola Nacional de Saúde Pública Sergio Arouca, Fundação Oswaldo Cruz, Rio de Janeiro, Brasil.; 2 Instituto Nacional de Câncer José de Alencar Gomes da Silva, Rio de Janeiro, Brasil.

Agradecemos às editoras pela oportunidade de contribuir com o debate sobre os cuidados paliativos, em especial no que tange à sua conceituação e aos fundamentos epistemológicos que sustentam sua aplicação em contextos clínicos e acadêmicos. 

Acolhemos com atenção a carta de Silva et al. ^1^, no entanto, consideramos necessário manifestar nossa discordância de afirmações que, em nossa análise, carecem de precisão conceitual e rigor metodológico. Assim, apresentamos os devidos esclarecimentos e contrapontos com base em análise documental de literatura científica amplamente reconhecida.

Nosso artigo, *Situações Difíceis e Sentimentos no Cuidado Paliativo Oncológico* ^2^, foi desenvolvido em unidade pública hospitalar exclusiva de cuidados paliativos oncológicos. O objetivo foi analisar situações difíceis e sentimentos vivenciados por profissionais de saúde no cuidado. O conceito de cuidados paliativos adotado segue a definição da Organização Mundial da Saúde (OMS) ^3^ ^,^ ^4^ ^,^ ^5^ e está em consonância com a recente Política Nacional de Cuidados Paliativos (PNCP) ^6^, sancionada após a publicação do estudo ^2^.

Silva et al. ^1^ (p. 1) afirmam que o conceito de “*cuidado paliativo se destina a todas as pessoas com quaisquer condições graves* ou *ameaçadoras ao longo de todos os ciclos de vida*” (grifo nosso). E acrescentam ^1^ que o conceito estaria plenamente estabelecido e “repousa sereno” sobre a resolução da 67ª Assembleia Mundial da Saúde ^5^. Contudo, essa definição dos autores não corresponde à redação oficial do documento, que mantém a referência a “doenças que ameaçam a vida”. Não há menção à expressão “condições ou doenças graves” ^4^ ^,^ ^5^, como detalhamos em nossa revisão conceitual das referências mencionadas na carta ^1^ e apresentadas no Quadro 1.

**Quadro 1:** Apresentação dos conceitos de cuidados paliativos em documentos oficiais do Ministério da Saúde, Organização Mundial da Saúde, Associação Internacional de Hospices e Cuidados Paliativos (IAHPC, acrônimo em inglês) e artigos científicos (período: 2002-2024).

CONCEITO DE CUIDADOS PALIATIVOS	AUTOR (ANO)
“*A definição anterior da OMS sobre cuidados paliativos destacava sua relevância para pacientes que não respondiam a terapias curativas. Essa afirmação poderia ser interpretada como relegando os cuidados paliativos às últimas etapas do tratamento. Hoje, no entanto,* há um amplo reconhecimento de que os princípios dos cuidados paliativos devem ser aplicados o mais cedo possível no curso de qualquer doença crônica e fatal” (p. 92, grifo nosso)	Sepúlveda et al. ^12^ (2002)
“*Os cuidados paliativos precisam começar cedo, durante a evolução da doença, para realmente melhorar a qualidade de vida e* o fim de vida dos pacientes” (p. 734, grifo nosso)	Temel et al. ^13^ (2010)
“*Considerando que os cuidados paliativos são uma abordagem que melhora a qualidade de vida de pacientes (adultos e crianças) e suas famílias que* enfrentam problemas associados a doenças ameaçadoras da vida*, através da prevenção e alívio do sofrimento por meio da identificação precoce e da avaliação e tratamento corretos da dor e outros problemas, sejam físicos, psicossociais ou espirituais*" (p. 1, grifo nosso) “...os cuidados de fim de vida para indivíduos estão entre os componentes críticos dos cuidados paliativos” (p. 2, grifo nosso)	Organização Mundial da Saúde ^5^ (2014)
“*Os cuidados paliativos são cuidados holísticos ativos, ofertados a pessoas de todas as idades que se encontram em intenso sofrimento relacionados à sua saúde, proveniente de doença severa,* especialmente aquelas que estão no final da vida” (p. 1, grifo nosso)	Associação Internacional de Hospices e Cuidados Paliativos ^7^ (2018)
“*Cuidados paliativos são o cuidado ativo e holístico de indivíduos de todas as idades com sofrimento grave relacionado à saúde, devido a doenças graves,* especialmente daqueles próximos do fim da vida” (p. 3, grifo nosso)	Radbruch et al. ^8^ (2020)
“*Cuidados paliativos são uma abordagem que promove a qualidade de vida de pacientes e suas famílias,* diante de doenças que ameaçam a continuidade da vida*, por meio da prevenção e alívio do sofrimento. Requerem identificação precoce, avaliação impecável e tratamento da dor e outros sintomas físicos, psicossociais, emocionais e espirituais, oferecidos por equipe multiprofissional, de forma integral e contínua, em todos os níveis de atenção à saúde*” (grifo nosso) “*Parágrafo 1º, Art. 1º da PNCP: Para os fins deste Anexo, compreende-se como cuidados paliativos as ações e os serviços de saúde para alívio da dor, do sofrimento e de outros sintomas em* pessoas que enfrentam doenças ou outras condições de saúde que ameaçam ou limitam a continuidade da vida” (grifo nosso) “*Princípio 1º, Art. 2º da PNCP: I -* valorização da vida e consideração da morte como um processo natural” (grifo nosso)	Ministério da Saúde ^6^ (2024)

PNCP: Política Nacional de Cuidados Paliativos .

Fonte: elaboração própria.

A carta omite, ainda, definições de cuidados paliativos respaldadas por fontes oficiais ^4^ ^,^ ^5^ ^,^ ^6^, e interpreta equivocadamente os marcos conceituais internacionais, implicando ausência de integridade científica. As principais instituições da área reiteram que os cuidados paliativos visam à qualidade de vida de pacientes e familiares diante de doenças que ameaçam a continuidade da vida ^3^ ^,^ ^4^ ^,^ ^5^ ^,^ ^6^ ^,^ ^7^ ^,^ ^8^ (Quadro 1).

Desde a primeira formulação do conceito pela OMS, em 1989, passando pelas revisões em 2002, 2014 e debates recentes na 77ª Assembleia Mundial da Saúde ^9^, o termo tem sido alvo de tensões conceituais entre especialistas ^8^.

Em 2018, a Associação Internacional de Hospices e Cuidados Paliativos (IAHPC, acrônimo em inglês) reuniu 450 especialistas de diversas regiões do mundo para revisar o conceito de cuidados paliativos utilizando o método Delphi. A discussão revelou divergências substanciais sobre os limites e a abrangência do conceito, evidenciando que ainda não há consenso pleno ^8^. As tensões entre uma abordagem ampliada, centrada no alívio de todo sofrimento (doenças graves), e outra mais restrita, voltada à terminalidade da vida, demonstram que o conceito está longe de alcançar uma unificação serena, como afirmada por Silva et al. ^1^.

A confusão apresentada pelos autores ^1^ parece decorrer da falta de distinção entre os níveis conceituais e práticos. A leitura atenta do artigo de Radbruch et al. ^8^ mostra que a controvérsia se concentra na aplicabilidade prática do conceito, defendendo que pessoas com doenças graves, ou populações vulnerabilizadas, se beneficiem da abordagem dos cuidados paliativos. Não há melindre relativo a termos que mencionem a finitude da vida, posto o reconhecimento da morte como processo natural, inclusive, por ser um princípio fundamental do próprio conceito ^4^ ^,^ ^6^.

Ressaltamos que o conceito proposto por Silva et al. ^1^ com uma conjunção alternativa como “*ou*” ‒ “*doenças graves*” ou “*ameaçadoras*” ‒ implica grave imprecisão, comprometendo sua aplicabilidade em políticas públicas, práticas clínicas e acadêmicas.

A alegação de que o uso da definição da OMS em nosso artigo “alimenta estigmas” ‒ incorretamente descritos como “*vieses*” pelos autores ^1^ (p. 1) ‒ revela uma interpretação simplificada da complexidade epistemológica envolvida. Nosso estudo ^2^, alicerçado pelas contribuições de Norbert Elias ^10^, demonstra que o afastamento da morte na sociedade ocidental contemporânea dificulta o trabalho em cuidados paliativos. Os profissionais entrevistados relataram sofrimento e impotência relacionado à dificuldade de lidar com a morte e com a hegemonia da lógica curativa em sua formação profissional ‒ uma realidade que atravessa o cotidiano dos cuidados paliativos ^2^.

A ausência de uma formação profissional que reflita sobre questões inerentes à finitude da vida humana compromete o bem-estar dos profissionais e dificulta a implementação efetiva dos cuidados paliativos, conforme salienta a OMS ^9^ e a PNCP ^6^. Estudo ^11^ também ressalta que a centralidade do paradigma curativo na medicina contemporânea condiciona negligência ao cuidado e à qualidade de vida de pessoas próxima à morte.

Em consonância com esses achados, a 77ª Assembleia Mundial da Saúde ^9^ pontuou, pela primeira vez, o esgotamento emocional de profissionais que atuam com cuidados paliativos ‒ fenômeno também identificado em nosso estudo ^2^.

Portanto, longe de “escamotear a morte”, como sugerido por Silva et al. ^1^, nosso artigo reforça justamente a centralidade da morte como processo natural, conforme orientações da OMS ^4^ e da PNCP ^6^.

A recomendação de início precoce dos cuidados paliativos é amplamente aceita ^3^ ^,^ ^4^ ^,^ ^5^ ^,^ ^6^ ^,^ ^7^ ^,^ ^8^ ^,^ ^9^ ^,^ ^11^ ^,^ ^12^ ^,^ ^13^. No entanto, Silva et al. ^1^ parecem confundir essa diretriz com a lógica da prevenção primária. Tal confusão desconsidera que os cuidados paliativos podem, simultaneamente, ter uma abordagem de prevenção terciária voltada à melhoria da qualidade de vida e incluir doenças incuráveis e potencialmente fatais ^12^ ^,^ ^13^. O início precoce, portanto, refere-se ao momento do diagnóstico de uma condição que ameaça a vida, não à sua ausência.

Por fim, destacamos um erro factual dos autores ^1^, ao citarem a *Portaria GM/MS nº 3.694*, de 22 de maio de 2024, como instituidora da PNCP. A referida Portaria na carta dos autores ^1^, na verdade, trata do repasse de recursos ao Município de Japeri e foi publicada em 3 de maio de 2024. A PNCP está formalmente instituída pela *Portaria GM/MS nº 3.681*, de 7 de maio de 2024 ^6^.

Reafirmamos que as definições adotadas em nosso trabalho estão fundamentadas em bases epistemológicas sólidas e alinhadas às diretrizes nacionais e internacionais. A fidelidade às fontes oficiais e à coerência conceitual é um dever ético na produção científica, especialmente em campos de debate como o dos cuidados paliativos. 

A integridade científica rejeita veementemente omissões deliberadas, interpretações distorcidas, ausência de contextualizações e leituras superficiais da literatura, tendo em vista que tais práticas comprometem a credibilidade da produção do conhecimento.

## References

[1] Silva AFS, Fonseca MRA, Cintra AC (2025). Imprecisão ameaçadora: superando vieses no conceito de cuidado paliativo.. Cad Saúde Pública.

[2] Beserra VS, Brito C (2024). Situações difíceis e sentimentos no cuidado paliativo oncológico. Cad Saúde Pública.

[3] World Health Organization (2021). Assessing the development of palliative care worldwide: a set of actionable indicators.

[4] World Health Organization (2002). National cancer control programmes: policies and managerial guidelines..

[5] World Health Organization (2014). Sixty-seventh World Health Assembly. Strengthening of palliative care as a component of comprehensive care throughout the life course..

[6] Ministério da Saúde (2024). Portaria GM/MS nº 3.681, de 7 de maio de 2024. Institui a Política Nacional de Cuidados Paliativos - PNCP no âmbito do Sistema Único de Saúde - SUS, por meio da alteração da Portaria de Consolidação GM/MS nº 2, de 28 de setembro de 2017.. Diário Oficial da União.

[7] International Association for Hospice and Palliative Care (2018). Global consensus based palliative care definition.

[8] Radbruch L, Lima L, Knaul F, Wenk R, Ali Z, Bhatnaghar S (2020). Redefining palliative care-a new consensus-based definition. J Pain Symptom Manage.

[9] World Health Organization (2024). Seventy-seventh World Health Assembly..

[10] Elias N (2001). A solidão dos moribundos, seguido de "envelhecer e morrer".

[11] Knaul FM, Farmer PE, Krakauer EL, Lima L, Bhadelia A, Kwete XJ (2018). Alleviating the access abyss in palliative care and pain relief-an imperative of universal health coverage the Lancet Commission report. Lancet.

[12] Sepúlveda C, Marlin A, Yoshida T, Ullrich A (2002). Palliative care the World Health Organization's global perspective. J Pain Symptom Manage.

[13] Temel JS, Greer JA, Muzikansky A, Gallagher ER, Admane S, Jackson VA (2010). Early palliative care for patients with metastatic non-small-cell lung cancer. N Engl J Med.

